# Retraction: Real-World Outcomes of Peri-Operative Chemotherapy With Fluorouracil, Leucovorin, Oxaliplatin, and Docetaxel in Upper Gastrointestinal Cancers: An Audit of Patients Diagnosed Between 2017 and 2020 at the Christie Hospital

**DOI:** 10.7759/cureus.r227

**Published:** 2026-04-16

**Authors:** Mariam Sabry, Yazan Mahmoud, Yass Hatahet, Rianne Patel, Nour Ibrahim

**Affiliations:** 1 Oncology Department, The Christie National Health Service (NHS) Foundation Trust, Manchester, GBR; 2 Oncology Department, University Hospitals Morecambe Bay, National Health Service (NHS), Morecambe Bay, GBR

The Editor-in-Chief has retracted this article at the request of the authors following concerns regarding the use of data. The data were collected as part of a formally registered internal hospital quality improvement and clinical audit, and it was subsequently recognized that appropriate permissions and acknowledgments related to the audit and its contributors were not fully addressed prior to publication. As these data are not in the public domain, the contents have therefore been redacted and the article retracted. The authors agree with the decision to retract.

